# Comparative evaluation of topical and intramuscular coenzyme Q10 application in non-surgical periodontal disease management: A randomized clinical study

**DOI:** 10.6026/973206300222174

**Published:** 2026-04-30

**Authors:** Shalini Sinha, Kausar Parwez Khan, Rupam Kumari Rupam, Ria Jha, Akshta Singh, K Mohamed Adhil

**Affiliations:** 1 Department of Periodontology and Implantology, Mithila Minority Dental College and Hospital, Darbhanga, Bihar, India; 2 Department of Periodontology, Vinayaka Mission's Sankarachariyar Dental College, Vinayaka Mission's Research Foundation (Deemed to be University), Salem, Tamil Nadu, India

**Keywords:** Coenzyme Q10, chronic periodontitis, scaling and root planing (SRP), intrasulcular drug delivery, topical application, periodontal therapy, antioxidants, probing pocket depth (PPD)

## Abstract

Periodontal disease remains challenging to manage because adjunctive antioxidant therapies are not well standardized forroute of administration or dosing relative to scaling and root planing (SRP). Therefore, it is of interest to evaluate SRP alone, SRP plus topical CoQ10 and SRP plus intrasulcular CoQ10 at 150 sites in 50 patients with chronic periodontitis over 4 weeks. Plaque Index, Gingival Index, Modified Sulcular Bleeding Index and Probing Pocket Depth were assessed at baseline and follow up in all three groups. All groups showed significant improvement, but Group 3 (intrasulcular CoQ10 + SRP) demonstrated the greatest reduction in probing pocket depth, from 6.52 ±1.07 mm to 3.67±0.28 mm (p = 0.000). Thus, intrasulcular CoQ10 + SRP is superior for periodontal regeneration opening new adjunctive antioxidant therapy pathways.

## Background:

Periodontitis is defined as chronic inflammation and overproduction of reactive oxygen species (ROS), resulting in tissue destruction, bone loss and loss of clinical attachment [1]. Oxidative stress, the imbalance between ROS and antioxidants, is a pathogenic factor in the development of periodontal disease [2]. Antioxidants such as Coenzyme Q10 (CoQ10) help restore redox balance, reduce oxidative impairment and support tissue healing [3]. Also, CoQ10 has been found to regulate immune function by reversing the effects of pro-inflammatory cytokines (TNF- α and IL-6) and enhancing anti-inflammatory cytokines (IL-10) and thereby facilitating wound healing and gingival regeneration [4, 5]. CoQ10, also called ubiquinone, is a naturally occurring, lipid-soluble compound that is concentrated in the human cell mitochondria [6]. It plays a central role in the electron transport chain during cellular respiration and helps produce ATP [7]. In addition to its role in bioenergetics, CoQ10 is a potent antioxidant [8]. CoQ10 in its reduced form (ubiquinol) removes ROS, thus avoiding oxidative damage to cellular components such as lipids, proteins and DNA [9]. Several researchers have reported markedly reduced CoQ10 levels in gingival tissues and serum of patients with chronic periodontitis, indicating a localised CoQ10 deficiency in the diseased areas [10]. This shortage can result from increased oxidative load, metabolic demand or defective biosynthesis [11]. CoQ10 supplementation has thus been suggested as a way of enhancing periodontal outcomes through strengthening the antioxidant defence system [12]. Therefore, it is of interest to determine whether intrasulcular or topically administered CoQ10 gel is an effective modality for alleviating the severity of chronic periodontitis in patients who have undergone SRP.

## Materials and Methods:

The interventional study was conducted in the Department of Periodontology at our institute. This research was checked and accepted by the Institutional Ethics Committee of the Mithila Minority Dental School (EC/NEW/INST/2023/4152) on May 5, 2023. The research was conducted from November 2023 to January 2025. The ethics of this study were consistent with the principles set out in the Declaration of Helsinki (2017). The sample size (n) was calculated using the following formula: n = 2(Zα)^2^ x SD^2^ / d^2^, Where: SD = expected standard deviation from previous research (10), Zα = standard normal variate at 5% level of significance (1.96), d = minimum expected difference in means (4), Substituting the values: n = 2 x (1.96)^2^ x (10)^2^ / (4)^2^, n = 2 x 3.84 x 100 / 16, n = 768 / 16 = 48. Thus, the calculated sample size was 48 participants. Taking into consideration a 10 per cent dropout rate, the sample was estimated to be 53. Nevertheless, the dropout rate decreased towards the end of the study and the resulting sample size was 50 patients. The sample of the study was selected based on clinical and radiographic criteria and comprised fifty patients having a history of chronic periodontitis and whose age ranged between thirty and sixty years. Such requirements included a probing pocket depth of at least 5 millimeters and bone loss in at least 3 quadrants. All patients were deemed in good health. The patients were not to include those with systemic diseases, those who have received antibiotic therapy in recent times or periodontal therapy, smokers, pregnant or lactating women. There were 150 study sites, with each participant randomly assigned to three quadrants and the study used computer- generated random numbers for various treatment protocols.

## The study consisted of three groups:

[1] Group 1: Scaling and Root Planing (SRP) only.

[2] Group 2: SRP with topical application of CoQ10 gel at a concentration of 10 mg/mL (Biocruz Pharmaceuticals (P) Limited and United Laboratories, IND).

[3] Group 3: SRP with intra-pocket application of CoQ10 gel.

## Treatment procedure:

Once the patients had been selected based on the above criteria and assigned to groups using a computer-generated algorithm, they were asked to be treated. The consent of all persons was obtained in writing after an explanation of the study details and its probable publication in a scientific journal. All the patients were asked to rinse their lips with Betadine mouthwash before scaling and root planing (SRP). The remaining two sites were thoroughly isolated from each other using cotton rolls and allowed to dry in the air once the SRP was over. The topical use of CoQ10 gel (10mg/mL) was applied to site 2. Using an applicator, the topical CoQ10 gel was applied. Applications were performed thrice at the site, once a week (Monday, Wednesday and Friday at 18:00 hours), with a 1-minute interval between applications. The entire gel application was performed by the dentist who performed the SRP. Site 3 was incubated with an intra- purpose application of CoQ10 gel, as in Site 2. The gel was delivered using an insulin needle. The gel was applied at the location as well as after each alternate day over a period of one week (Monday, Wednesday and Friday at 18:00 hours). The clinical parameters recorded were the Modified Sulcular Bleeding Index, Probing Depth, Gingival Index and Plaque Index, the baseline and four-week treatment times recorded with the UNC-15 probe. Baseline data of each patient was taken during the first visit; the Modified Sulcular Bleeding Index (Newbrun [13], Probing Depth with UNC-15 probe, Gingival Index (Loe, 1967) [14] on four surfaces: Dentofacial papilla, facial margin, mesiofacial papilla and lingual margin and Plaque Index (Loe, 1967) [14] on mesial, buccal, distal and lingual surfaces. A blind, pretrained examiner recorded all the data. These parameters were retested four weeks after treatment on the same teeth. Altered Sulcular Bleeding Index rates the location based on bleeding reaction following soft probing of the 4-point, also-rated, sulcus or peri-implant pocket on a 4-point (0 to 3) scale. The meaning of no bleeding (Score 0) is to be healthy, whereas the meanings of 1, 2 and 3 give slight, moderate and severe inflammation, respectively. Probing Depth is the distance between the top of the gingival margin and either/both: the base of a periodontal pocket or the bottom of the gingival sulcus. A periodontal probe is an instrument used to measure the depth. Six locations are measured around each tooth (or implant) and the findings will be documented to monitor disease progression and the success or failure of periodontal treatment in the long run. A widely used clinical index to quantitatively assess the severity of gingival inflammation is the Gingival Index, developed in 1963 [14]. It scores the gingiva based on colour, consistency and bleeding on gentle probing. Plaque Index was specifically designed to assess plaque in the gingival region, which is most important in initiating gingivitis. The index uses a graded scale of 4 (0-3), where a lack of detectable plaque is graded 0 and soft debris is graded 1-3. A dental probe may be used to make plaque visible by moving it over the tooth surface, or a disclosing agent can be used. Plaque Index is significant in assessing a patient's oral health and the effectiveness of brushing and flossing.

## Statistical analysis:

All the observed data were organized in an Excel sheet. T-tests and ANOVA were conducted using SPSS (v21, Windows, IBM and CL, USA).

## Results:

The Results show that 33 (65%) of patients were aged 30-40, 12 (25%) were aged 41-50 and 5 (10%) were aged 51 and above. Thirty participants were identified as male, 20 as female, for a total of 50 participants of both genders. Table 1 (see PDF) compares Plaque Index scores between groups over time. Group 1 significantly reduced from 2.2400 ± 0.46686 at baseline to 1.4600 ± 0.27899 at 4 weeks (p = 0.003). Groups 2 and 3 also showed significant decreases from 2.2400 ± 0.50049 to 1.1820 ± 0.27899 and from 2.0900 ± 0.48792 to 0.4960 ± 0.23296, respectively (both p = 0.001). The overall p-value was 0.119 at baseline (not significant) and 0.000 at 4 weeks (highly significant). Table 2 (see PDF) shows a significant reduction in Gingival Index scores across all groups after 4 weeks. Group 1 decreased from 1.6940 ± 0.44420 to 0.7060 ± 0.22983, Group 2 from 1.8920 ± 0.47631 to 0.5220 ± 0.21880 and Group 3 from 1.8540 ± 0.47518 to 0.6240 ± 0.20855 (all p = 0.001). The overall p-value was 0.082 at baseline (not significant) and 0.000 at 4 weeks (highly significant). Table 3 (see PDF) compares Modified Sulcular Depth between groups over time. Group 1 showed a significant reduction from 1.9200 ± 0.56928 at baseline to 1.0100 ± 0.41711 at 4 weeks (p = 0.003). Group 2 decreased from 1.9160 ± 0.54562 to 1.1980 ± 0.27957 (p = 0.04) and Group 3 decreased from 1.9000 ± 0.52722 to 1.0020 ± 0.44561 (p = 0.02), both statistically significant. The overall p-value was 0.098 at baseline (non-significant) and 0.001 after 4 weeks (significant). Table 4 (see PDF) compares Probing Pocket Depth between groups over time. Group 1 showed a significant reduction from 5.1760 ± 0.94211 at baseline to 3.6460 ± 0.59803 at 4 weeks (p = 0.000). Group 2 decreased from 5.6380 ± 1.06117 to 3.6400 ± 0.57143 and Group 3 from 6.5160 ± 1.06663 to 3.6740 ± 0.61072 (both p = 0.000), indicating highly significant improvements. The overall p-value was 0.958 at baseline (non-significant) and 0.000 at 4 weeks (highly significant).

## Discussion:

Contemporary science has made significant progress in the treatment and perception of periodontal disease. Scaling and Root Planing (SRP) is still considered the main pillar of non-surgical periodontal therapy, aimed at clearing biofilm and calculus [15]. Nonetheless, there have been recent developments in adjunctive treatments, especially those that use antioxidants, such as CoQ10, which may help regulate the host response and ROS [16]. Chronic periodontitis is an inflammatory disease triggered by the plaque biofilm and driven by an imbalance between ROS and antioxidant defences [17]. Plaque biofilm initiates inflammation, which destroys the periodontal ligament and alveolar bone, hence characterising chronic periodontitis [18]. The disease is associated with an imbalance between the antioxidant defence system and ROS. ROS that are needed only in small amounts during normal cell signalling can cause severe tissue damage when overproduced. Oxidative stress leads to the development of periodontal disease [19]. Littarru *et al.*. [20], Manthena *et al.*. [21] and Golafrouz *et al.*. [22] have concluded that there is some connection between periodontal disease and CoQ10 deficiency. CoQ10, the reduced form (ubiquinol), is a good antioxidant and has demonstrated the potential to repair tissues and alleviate inflammation. Endogenous production, as well as dietary sources, can be used to obtain it and its safety profile is high [23]. CoQ10 is an antioxidant, which is naturally present in reduced form (ubiquinol) and is important in energy production in the mitochondria [24]. It also prevents the oxidative damage of lipids and cell membranes [25]. Its exogenous and endogenous availability, along with its good safety profile, make it appropriate for systemic and local treatments [26]. Delivered, e.g., gels have the benefit of very high drug concentrations at the target site and minimal systemic exposure, minimizing side effects and increasing patient compliance [27]. In this case, we considered three conditions: SRP without any other conditions (control), SRP with topical CoQ10 use and SRP with CoQ10 applied intra-pocket. When Group 2 and Group 3 were compared with Group 1, it was found that the plaque, gingival and bleeding indices significantly decreased. Clinical efficacy between Group 2 (topical application) and Group 3 (intra-pocket application) differed significantly. Groups 2 and 3 also exhibited a significant change in probing pocket depth compared to the control group, indicating that CoQ10 use, along with the treatment, enhanced healing and tissue response. These findings are consistent with those of Hanioka *et al.*. who reported better gingivitis and fewer bleeding on probing with subgingival CoQ10 application [28]. Farahmand *et al.*. also found improved results under CoQ10 therapy in combination with oral hygiene [10]. On the other hand, Dahiya *et al.*. did not statistically demonstrate a difference between SRP and CoQ10 gel, alone or in combination with CoQ10, indicating that gel form and method of delivery could affect the clinical effect of CoQ10 [29]. The findings of the current study are supported by Dahiya *et al.*. [29], who conducted a split-mouth trial using an intra-pocket CoQ10 gel and found significant improvements in periodontal parameters without the use of a periodontal dressing. Additionally, Shaheen *et al.*. [30] demonstrated that CoQ10 in a nano-formulation was effective, indicating that formulation plays a crucial role in bioavailability and retention in the periodontal pocket. Systemic CoQ10 has also shown promise. Manthena *et al.*. [21] and Prakash *et al.*. [31] demonstrated a considerable improvement in both gingival inflammation and clinical parameters with systemic supplementation and also concluded that CoQ10 can improve the outcomes of non-surgical periodontal treatment when taken long-term as a dietary supplement in both local and systemic forms. The findings of the current research indicated that intra-pocket CoQ10 gel application was the most effective in clinical terms, implying increased retention and efficacy when applied directly to the periodontal pocket. Substantivity and localised action on antioxidants were high, which may have contributed to the better results in this group. Nonetheless, conclusions regarding long-term benefits are not possible given the short duration of the study. This clinical trial aimed to compare the performance of SRP with that of SRP in combination with a topical or intra-pocket CoQ10 gel. Group 2 and Group 3 (CoQ10-treated) showed a considerable reduction in the plaque index, gingival index and bleeding index compared to Group 1. There were greater improvements in the intra-pocket delivery of CoQ10 compared to topical application, especially in Groups 2 and 3. The probing pocket depth also significantly increased in both Group 2 and Group 3, indicating improved periodontal healing. Past research has given inconsistent findings. Although they were not statistically significant in the studies by Sharma *et al.*. [32] and Barakat and Attia [33], respectively, when compared to CoQ10, others, e.g., those by Shaheen *et al.*. reported better results with nano-formulated CoQ10 [30]. Hanioka *et al.*. reported that topical CoQ10 use improved gingival health. Nevertheless, he did not find a considerable difference between SRP and SRP + Perio-Q gel [28]. Worthy of mention, though, are the findings of Dahiya *et al.*., which heavily favored the use of CoQ10 gel within the intra-pocket without any packing, which correlates with the results of this paper [29]. CoQ10 Systemic CoQ10 supplementation has also been examined. Studies by Manthena *et al.*. [21] and Prakash *et al.*. [31] confirmed that oral CoQ10 had a significant effect on periodontal parameters and concluded that dietary supplementation used over an extended time may have a positive effect on non-surgical periodontal therapy. The current research revealed that adjunctive CoQ10 therapy, particularly intra-pocket application, offers significant clinical advantages compared to SRP alone. This could be due to increased localised antioxidant activity, improved substantivity and increased penetration into the periodontal pocket. Nevertheless, there are also limitations, such as the limited follow-up period. There is a need to research the long- term effects and the substantivity of the gel.

## Conclusion:

CoQ10 when delivered intra-pocket (as a gel), improves the clinical efficacy of SRP in the treatment of chronic periodontitis. It helps alleviate inflammation, clinical indices and periodontal healing. Although there are encouraging short-term outcomes, more datawith longer follow-up, larger sample sizes and standardised delivery options is required to demonstrate the long-term effects and sustainability of CoQ10 gel in periodontal therapy.
